# Lifestyle intervention improves cardiometabolic profiles among children with metabolically healthy and metabolically unhealthy obesity

**DOI:** 10.1186/s13098-024-01493-8

**Published:** 2024-11-11

**Authors:** Ruziana Mona Wan Mohd Zin, Muhammad Yazid Jalaludin, Fuziah Md Zain, Janet Yeow Hua Hong, Nur Zati Iwani Ahmad Kamil, Abdul Halim Mokhtar, Wan Nazaimoon Wan Mohamud

**Affiliations:** 1 Endocrine and Metabolic Unit, Institute for Medical Research, National Institutes of Health, Ministry of Health Malaysia, Setia Alam, Selangor Malaysia; 2 Department of Paediatrics, Faculty of Medicine, Universiti Malaya, Kuala Lumpur, Malaysia; 3 Department of Paediatrics, Hospital Putrajaya, Ministry of Health Malaysia, Putrajaya, Malaysia; 4 Unit of Sports Medicine, Faculty of Medicine, Universiti Malaya, Kuala Lumpur, Malaysia

**Keywords:** School children, Paediatric obesity, Cardiometabolic risk factors, Lifestyle intervention, School-based intervention, Metabolic phenotype

## Abstract

**Background:**

In recent years, there has been a surge of interest in the metabolic phenotype among children with obesity characterized by the absence of associated cardiometabolic risk factors (CRFs), known as metabolically healthy obesity (MHO), as opposed to those with metabolically unhealthy obesity (MUO). This study investigated the effect of lifestyle intervention on CRFs among children with MHO and MUO.

**Methods:**

A total of 102 school-aged children with obesity (54 girls and 48 boys) aged 8–16 years completed a 16-week school-based lifestyle modification intervention program, MyBFF@school Phase I. The intervention consisted of physical activity, healthy eating promotion, and psychological empowerment. MHO and MUO statuses were defined based on the 2018 consensus-based criteria. Fasting venous blood collection, body composition measurement, clinical assessment and physical fitness testing were conducted at baseline and at the end of week 16.

**Results:**

After the intervention, the CRFs of the children with MUO improved with significant decreases in systolic (*p* < 0.001) and diastolic (*p* = 0.01) blood pressure and a significant increase in high-density lipoprotein cholesterol (HDL-C) (*p* = 0.005), while the CRFs of the children with MHO had a significant decrease in uric acid (*p* = 0.04). Additionally, 51.6% of the children with MHO transitioned to the MUO, while 26.8% of the children with MUO crossed over to the MHO at the end of the intervention. Furthermore, the odds of having high systolic blood pressure among children with MUO were 59% lower at week-16 than at baseline (OR = 0.41 (95% CI = 0.18, 0.92), *p* = 0.03).

**Conclusions:**

Our findings demonstrated that CRFs improved more prominently among children with MUO following the intervention. More importantly, our findings indicate that MHO in children is transient, hence, strategies to protect children against MUO are warranted.

*Trial registration*: ClinicalTrials.gov NCT02212873.

## Introduction

The incidence of childhood obesity is on the rise in many countries around the world [1], and childhood obesity has been linked to a variety of chronic diseases, including type 2 diabetes mellitus, dyslipidaemia, hypertension, and fatty liver disease [2]. In Malaysia, national surveys indicate that the prevalence of childhood obesity increased substantially from 11.9% in 2015 [3] to 14.8% in 2019 [4]. Children with obesity are at an increased risk of adult mortality [5], making it one of the most concerning public health issues. However, evidence shows that not all individuals with obesity experience the same level of obesity-associated complications. In recent years, there has been a surge of interest in the metabolic phenotype among children with obesity characterized by the absence of associated cardiometabolic risk factors (CRFs), known as metabolically healthy obesity (MHO). Children with MHO have normal blood lipid, glucose, and blood pressure levels, as opposed to those with metabolically unhealthy obesity (MUO) [6].

Identifying MHO in children is crucial for understanding the mechanisms that guard against the clustering of CRFs. Subsequently, rather than one-size-fits-all obesity management, a clear differentiation between metabolic phenotypes could be advantageous in delivering more effective and targeted treatment for children with obesity [7]. Currently, there are no universally accepted criteria for classifying MHO in children, although many previous studies have used various cut-off values for metabolic syndrome components and insulin sensitivity to define MHO in children [8–12]. Owing to this, Damanhoury et al. [13] proposed the first international consensus-based definition of MHO for the paediatric population in 2018, and this attempt is vital to limit heterogeneity in MHO definitions and for facilitating cross-study comparisons [11].

Necessary measures are needed to stop the deterioration in cardiometabolic function and to reduce the risk of developing chronic diseases associated with obesity in children with both MHO and MUO. However, treating obesity is time-consuming and complex and often fails to achieve the desired weight loss goals and health outcomes [14]. Several studies on lifestyle modification interventions for obesity, which incorporated multiple components such as physical activity, diet, and behavioural therapy, have shown significant improvements in as little as 3 months [15, 16]. Additionally, studies have reported that children playing small-sided football games tend to have higher heart rates compared to those participating in larger-sided games, with effects similar to those of high-intensity interval training [17, 18]. Furthermore, increased cardiorespiratory fitness through physical activity has been linked to the MHO phenotype in adults [19].

In adults, lifestyle interventions have been reported to have health benefits for both MHO and MUO, although greater metabolic improvement was found among adults with MUO despite similar weight loss [20]. However, evidence for children with MHO and MUO following lifestyle intervention is still lacking, hence, our findings add to the current literature by reporting the effect of lifestyle intervention on CRFs and blood profiles related to obesity among children with MHO and MUO.

## Materials and methods

### Study participants

**Fig. 1:**
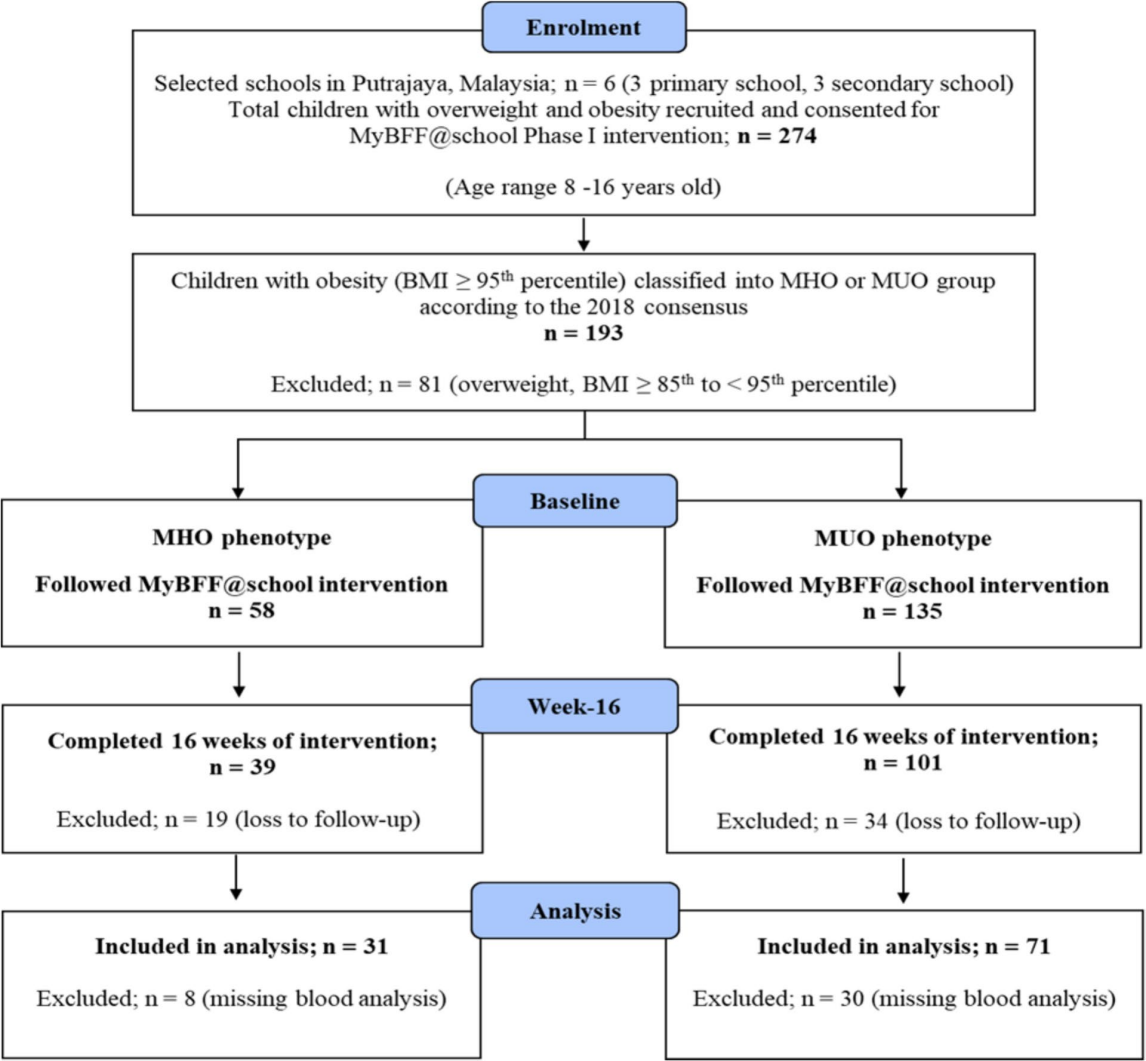
Flow chart of the MyBFF@school Phase I intervention and the final MHO and MUO analyses

We utilised G*Power 3.1 software to calculate the necessary sample size to evaluate the mean differences across all parameters at baseline and week-16 for both the metabolically healthy obesity (MHO) and metabolically unhealthy obesity (MUO) groups. The analysis employed *F*-tests for repeated measures ANOVA (within-subjects factors). The following parameters were specified for the calculation; effect size (partial eta-squared) = 0.02, nonsphericity correction = 1, correlation among repeated measures = 0.5, number of groups = 2, number of measurements = 2, significance level (α) = 0.05 and desired power = 80%. Based on these parameters, the minimum required sample size was determined to be 100 participants. A total of 102 school-aged children (54 girls and 48 boys) aged 8–16 years were recruited for this study and completed the 16 weeks of lifestyle intervention in 2014 (Fig. 1). The “My Body is Fit and Fabulous at School Phase I (MyBFF@school)” was a school-based lifestyle intervention study specifically intended for schoolchildren with obesity to help them lose weight. The MyBFF@school is a multifaceted obesity intervention program that incorporates physical activity, healthy eating promotion, and psychological empowerment and was conducted in 3 primary and 3 secondary government schools in Putrajaya, Malaysia. Children with obesity who were diagnosed with either physical or medical conditions that prevented them from participating in moderate-to-vigorous physical activity were excluded from this study. In addition, those who were diagnosed with comorbidities that may interfere with the analysis such as congenital heart disease, renal, hepatic, or endocrine diseases related to obesity, were also excluded from this study. Parents and children provided written informed consent and assent, and all tests were carried out following the approved guidelines. Those excluded from the study continued to receive the standard health education program offered by their school. Additionally, children with underlying health conditions were referred to nearby clinics or hospitals for more effective monitoring. The study protocol was reviewed and approved by the Medical Research and Ethics Committee, Ministry of Health Malaysia, and the methodology was previously described in detail [21]. This study was also registered with ClinicalTrials.gov (identifier NCT02212873).

### Intervention protocol

Participants in this study were required to participate in the MyBFF@school Phase I intervention program, which included physical activity, healthy eating promotion, and psychological empowerment, for 16 weeks. The physical activity component was delivered in the form of a small-sided football game (SSG) three times a week for 1 h per session. The physical activity sessions were held on school days in the evening, from 4:00 PM to 4:45 PM, after classes had ended. Children were grouped into teams of 4 (minimum) to 7 (maximum) players each, with teams separated by gender. They began with 10 min of warm-up which included stretching, ball kicking, and dribbling. The SSG football game took up the following 45 min, with a 5-min half-time break. After the games, the last 5 min were spent cooling down. Each SSG game was played in a small field of approximately 14 × 9 m and was supervised by a trained coach who encouraged active participation from all players. The SSG game was designed to promote greater interaction, involvement, and movement among the children, thereby maximizing their physical activity levels during play. For example, one of the game’s rules requires that all players on the same team must touch the ball at least once before scoring.

The nutritional sessions were held twice a month for 45 min per session to promote healthy eating behaviours among the children. Nutritional education was provided in the classroom and complemented with interactive activities to strengthen the children’s nutritional knowledge, attitudes, and practices. The interactive activities include a nutrition-themed board game, interactive quizzes, role-playing, and healthy cooking demonstrations, all designed to be both educational and enjoyable for the children. A team of nutritionists provided group nutritional counselling for the children and their parents/guardians in four sessions throughout the study period to promote healthy eating at home. Finally, the psychological session was held twice a month for 45 min each. The participants gathered in a single classroom, where they were guided by a team of clinical psychologists. To enhance engagement, the psychological lectures were supplemented with activities such as quizzes, storytelling, and role-playing. Psychological empowerment education aimed to improve self-esteem, social skills, assertiveness, and positive thinking among children with obesity. To guarantee uniformity in carrying out the intervention, all intervention components were carried out by trained personnel.

### Physical and clinical assessments

The day before the study visit, the children were reminded to fast overnight for at least 8 h. The assessment began early in the morning at 7 AM and continued until approximately 12 PM. During this period, blood samples were collected, and body composition was measured while participants were still fasting. Following these procedures, participants were examined by medical doctors and then underwent a physical fitness test. Afterwards, they were divided into several classrooms to complete questionnaires. No physical activity, nutrition, or psychological sessions were conducted on assessment days. Standing height was measured to the closest 0.1 cm without wearing shoes using a calibrated stadiometer (Seca 217, Germany). Body weight, body fat mass, and skeletal muscle mass (SMM) were recorded to the nearest 0.1 kg in light clothing without shoes and socks using a bioelectrical impedance analyser (InBody 720, Korea). Body mass index (BMI) was calculated as weight in kilograms (kg) divided by the square of height in meters (m^2^). Waist circumference was measured twice to the precise 0.1 cm over the skin halfway between the tenth rib and the iliac crest at the end of normal respiration using an inelastic measuring tape (Seca 201, Germany). Systolic blood pressure (SBP) and diastolic blood pressure (DBP) were recorded twice on the right arm after 5 min of rest while the children were in a seated position using an appropriately sized cuff mercury sphygmomanometer (Accoson, UK). A pictorial Tanner staging scale was used for children to self-assess their pubertal stage [22, 23]. The modified Harvard Step Test was used to assess the physical fitness score (PFS) of the children by stepping up and down a 30 cm platform [24]. One of the children’s fingers was attached to a finger pulse oximeter, which continuously monitored the pulse rate and oxygen saturation (SpO_2_). The children were asked to step on and off the platform while following the pace of a metronome set at 120 beats per min for 5 min. Those with heart rates greater than 200 beats per min, SpO_2_ levels less than 90%, or inability to finish due to difficulty in breathing were promptly stopped. Immediately after they completed the 5 min or were stopped halfway due to the aforementioned reasons, the children needed to quickly sit down and rest on the platform. Heart rate was recorded at 0, 1, and 2 min of rest, as well as the total duration of exercise to the precise seconds. The PFS score was calculated by dividing the total duration of exercise in seconds by the sum of three heart rate measurements at 0, 1, and 2 min of rest.

### Biochemical measurements

Fasting venous blood was drawn from the children’s arms by experienced nurses and medical doctors. Blood samples were always kept cold at the study sites and processed within 2 h of being collected at the Institute for Medical Research central laboratory. Blood serum/plasma aliquots were stored at −20 °C for short-term storage or −80 °C for long-term storage before laboratory analysis. Fasting plasma glucose (FPG), total cholesterol (TC), triglycerides (TG), high-density lipoprotein-cholesterol (HDL-C), low-density lipoprotein-cholesterol (LDL-C), apolipoprotein A-1 (Apo A-1), apolipoprotein B (Apo B), high-sensitivity C-reactive protein (hsCRP), adiponectin, and uric acid were measured using an automated analyser (Dirui CS-400, China) with reagents purchased from Randox Laboratories (Antrim, UK). An automated enzyme immunoassay analyser (TOSOH AIA-360, Japan) was used to measure fasting insulin levels. Interleukin-6 (IL-6) concentrations were measured by an enzyme-linked immunosorbent assay (ELISA) using a commercially available kit with a sensitivity of 0.4 pg/mL (R&D Systems, USA), and the intra-and inter-assay coefficients of variation were less than 10%.

### Definitions of measures

Obesity was defined as a BMI for age and gender at or above the 95th percentile (BMI ≥ 95th percentile) according to the WHO 2007 growth chart [25]. Children with severe obesity were further classified as class 2 obesity (BMI ≥ 120% to <140% of the 95th percentile or BMI ≥ 35 kg/m^2^ to <40 kg/m^2^, whichever is lower based on age and gender) or class 3 obesity (BMI ≥ 140% of the 95th percentile or BMI ≥ 40 kg/m^2^, whichever is lower based on age and gender) according to the American Academy of Paediatrics 2023 clinical practice guidelines [26]. All children in this study were classified as having either MHO or MUO using the consensus-based classification proposed by Damanhoury et al. [13]. Children were classified as having MHO if they fulfilled all the following criteria: HDL-C > 1.03 mmol/L, TG ≤ 1.7 mmol/L, SBP and DBP ≤ 90th percentile, and FPG ≤ 5.6 mmol/L. Given that no agreement was achieved for the glycaemic level, a FPG ≤ 5.6 mmol/L was used because it was the most widely used parameter in prior studies of MHO in children [27]. Children with obesity who failed to fulfil one or more of the above criteria were categorized as having MUO. Tanner stage 1 external genitalia development for boys and breast development for girls were defined as prepubertal, whereas stage 2 and above were defined as pubertal. A waist circumference ≥90th percentile was defined as abdominal obesity according to the Malaysian children’s chart [28]. The Homeostasis Model Assessment of Insulin Resistance (HOMA-IR) index was determined as previously published [29]. The quantitative insulin sensitivity check index (QUICKI) was calculated as 1/[log fasting insulin (µU/mL) + log fasting glucose (mmol/L) × 18] [30]. The systolic and diastolic blood pressure percentiles were calculated according to the clinical practice guidelines for screening and management of high blood pressure in children and adolescents [31]. For children aged 10–16 years, metabolic syndrome was diagnosed based on the International Diabetes Federation recommendations [32] where it was considered present if the waist circumference was ≥ 90th percentile according to the Malaysian children’s chart [28], combined with at least 2 of the following criteria: HDL-C < 1.03 mmol/L, TG ≥ 1.7 mmol/L, FPG ≥ 5.6 mmol/L and SBP ≥ 130 mmHg and/or DBP ≥ 85 mmHg. Children aged below 10 years who fulfilled these criteria were classified as at risk for metabolic syndrome.

### Statistical analysis

The Kolmogorov–Smirnov test was used to test the normality of continuous variables. Continuous variables are presented as the mean and standard deviation (sd), and the differences between metabolic phenotypes at baseline were compared using the independent *t* test. Categorical variables at baseline are presented as frequencies and proportions, and comparisons between groups were made using the chi-square test. Mean differences within groups and between groups before and after intervention were analysed using repeated measures analysis of variance (ANOVA) adjusted for confounders and covariates. The odds ratios (ORs) and 95% confidence intervals (95% CIs) of the CRFs before and after intervention were determined using generalized estimating equations (GEEs). All analyses were carried out using IBM SPSS 28.0, with 2-sided *p* value <0.05 indicating statistical significance.

## Results

The final analysis included 102 Malaysian school-aged children with obesity aged 8–16 years old who completed the MyBFF@school Phase I intervention study (Fig. 1). At baseline, all our participants exhibited abdominal obesity, 64.7% were classified as severe obesity (class 2 and 3 obesity) and 30.4% had MHO. In addition, 14.8% of our participants had metabolic syndrome, whereas 9.5% were at risk for metabolic syndrome (children below 10 years old). The mean values of the criteria used to classify MHO or MUO were significantly different between the two groups at baseline (Table 1).

**Table 1:** Comparison of anthropometric data and parameters used as criteria to define MHO and MUO at baseline

	MHO	MUO	*p* value	Total
	*n* = 31 (30.4%)	*n* = 71 (69.6%)		*n* = 102 (100%)
Age (year), mean (sd)	11.8 (2.3)	12.3 (2.1)	0.26	12.1 (2.2)
BMI (kg/m^2^), mean (sd)	28.6 (3.7)	29.4 (3.8)	0.36	29.1 (3.8)
BMI *z*-score, mean (sd)	2.83 (0.59)	2.84 (0.53)	0.95	2.84 (0.54)
Weight category, *n* (%)				
Class 1 obesity	10 (32.3)	26 (36.6)	0.68	36 (35.3)
Class 2 obesity	14 (45.2)	34 (47.9)		48 (47.1)
Class 3 obesity	7 (22.6)	11 (15.5)		18 (17.6)
Gender, *n* (%)				
Girls	20 (64.5)	34 (47.9)	0.12	54 (52.9)
Boys	11 (35.5)	37 (52.1)		48 (47.1)
Pubertal status, *n* (%)				
Prepubertal	16 (51.6)	26 (36.6)	0.16	42 (41.2)
Pubertal	15 (48.4)	45 (63.4)		60 (58.8)
MHO/MUO criteria, mean (sd)				
FPG (mmol/L)	5.17 (0.32)	5.35 (0.52)	**0.04**	5.30 (0.42)
HDL-C (mmol/L)	1.24 (0.17)	1.09 (0.22)	**<0.001**	1.14 (0.22)
TG (mmol/L)	0.92 (0.32)	1.20 (0.50)	**0.005**	1.11 (0.46)
SBP (mm Hg)	102.97 (9.04)	110.32 (11.06)	**0.002**	108.09 (10.98)
DBP (mm Hg)	63.35 (6.53)	71.18 (9.19)	**<0.001**	68.80 (9.18)
HOMA-IR, mean (sd)	3.75 (2.06)	4.29 (2.56)	0.30	4.12 (2.42)
PFS, mean (sd)	58.0 (17.8)	55.3 (20.6)	0.53	56.1 (19.7)

The data are expressed as the mean (standard deviation) for continuous variables and as the proportion (%) for categorical data. Comparisons between groups were analysed using independent *t* tests and chi-square tests for continuous and categorical data respectively. Bold *p *values indicate statistical significance

*MHO* metabolically healthy obesity, *MUO* metabolically unhealthy obesity, *BMI* body mass index, *FPG* fasting plasma glucose, *HDL-C* high-density lipoprotein cholesterol, *TG* triglycerides, *SBP* systolic blood pressure, *DBP* diastolic blood pressure, *HOMA-IR* homeostasis model assessment of insulin resistance, *PFS* physical fitness score, *sd* standard deviation

Table 2 shows the changes in obesity-related anthropometry and blood profiles after 16 weeks of intervention. In terms of anthropometric alterations, both the MHO and MUO groups exhibited significant reductions in BMI and body fat percentage, as well as increases in SMM and PFS. However, the waist circumference of the MUO group increased significantly after the intervention (mean difference of 1.78 (95% CI: 0.60, 2.96), *p* < 0.001), whereas the waist circumference of the MHO group did not change. The MUO group exhibited improved CRFs after the intervention, with a significant decrease in SBP (*p* < 0.001) and DBP (*p* = 0.01), and a significant increase in HDL-C (*p* = 0.005). In contrast, the MHO group had a significant increase in DBP (*p* = 0.009) and TG (*p* = 0.02). Compared to those in the MUO group, the MHO group exhibited a substantially lower ratio of Apo B/Apo A-1 post-intervention (mean difference of −0.08 (95% CI: −0.15, −0.02), *p* = 0.02), despite both groups having a significant reduction in Apo A-1 and Apo B. Furthermore, compared with those in the MUO group, the MHO group exhibited a significantly higher level of adiponectin post-intervention (mean difference of 1.36 (95% CI: 0.39, 2.34), *p* = 0.007). In terms of changes in low-grade inflammatory markers following the intervention, the MHO group showed a significant decrease in uric acid (*p* = 0.04), even though both groups had significantly increased interleukin-6 (*p* < 0.001). For the insulin resistance markers, there were increasing trends in HOMA-IR and the TG: HDL-C ratio in both groups after the intervention, although these changes were not statistically significant.

**Table 2:** Changes in body composition and blood parameters in the MHO and MUO groups after the intervention

Status at baseline	Baseline	Week-16	Changes within group (week-16, baseline)		Changes between group (MHO, MUO)	
	Mean (95% CI)	Mean (95% CI)	Mean diff (95% CI)	*p* value	Mean diff (95% CI)	*p* value
*Body composition*						
BMI (kg/m^2^)^a^ (*n* = 102)						
MHO	29.0 (28.0, 30.1)	28.7 (27.6, 29.7)	−0.37 (−0.67, −0.06)	**0.02**	−0.15 (−1.43, 1.13)	0.82
MUO	29.3 (28.6, 29.9)	28.9 (28.2, 29.5)	−0.39 (−0.59, −0.19)	**<0.001**		
PBF (%)^a^ (*n* = 102)						
MHO	43.4 (41.7, 45.1)	41.9 (40.1, 43.6)	−1.54 (−2.39, −0.69)	**<0.001**	0.83 (−1.21, 2.86)	0.42
MUO	42.2 (41.1, 43.3)	41.3 (40.1, 42.4)	−0.93 (−1.49, −0.37)	**<0.001**		
WC (cm)^a^ (*n* = 102)						
MHO	88.0 (85.3, 90.7)	89.2 (86.5, 91.9)	1.17 (−0.69, 3.02)	0.21	−0.36 (−3.55, 2.83)	0.82
MUO	88.3 (86.5, 90.0)	90.0 (88.3, 91.8)	1.78 (0.60, 2.96)	**0.004**		
SMM (kg)^a^ (*n* = 102)						
MHO	19.6 (18.6, 20.6)	20.3 (19.2, 21.4)	0.74 (0.43, 1.05)	**<0.001**	−0.78 (−2.08, 0.52)	0.23
MUO	20.5 (19.8, 21.2)	21.0 (20.3, 21.7)	0.51 (0.30, 0.71)	**<0.001**		
*Fitness*						
PFS^a^ (*n* = 95, missing, *n* = 7)						
MHO	58.7 (50.9, 66.5)	68.5 (62.3, 74.6)	9.78 (0.62, 18.95)	**0.03**	−0.34 (−6.75, 6.06)	0.92
MUO	55.3 (50.1, 60.5)	72.6 (68.5, 76.7)	17.33 (11.19, 23.45)	**<0.001**		
*CRFs*						
SBP (mmHg)^b^ (*n* = 102)						
MHO	103.3 (99.9, 106.7)	104.3 (100.9, 107.7)	0.98 (−3.08, 5.04)	0.63	−3.34 (−6.58, −0.09)	**0.04**
MUO	109.7 (107.6, 111.8)	104.6 (102.4, 106.7)	−5.20 (−7.74, −2.66)	**<0.001**		
DBP (mmHg)^b^ (*n* = 102)						
MHO	64.7 (61.9, 67.4)	69.8 (66.6, 73.0)	5.09 (1.28, 8.90)	**0.009**	−2.02 (−4.71, 0.68)	0.14
MUO	70.9 (69.1, 72.6)	67.6 (65.6, 69.7)	−3.24 (−5.70, −0.79)	**0.01**		
FPG (mmol/L)^c^ (*n* = 102)						
MHO	5.18 (4.74, 5.61)	5.38 (5.15, 5.61)	0.20 (−0.19, 0.60)	0.08	−0.19 (−0.37, −0.01)	**0.04**
MUO	5.35 (5.23, 5.47)	5.59 (5.44, 5.73)	0.24 (0.08, 0.40)	**0.01**		
TG (mmol/L)^c^ (*n* = 102)						
MHO	0.91 (0.75, 1.08)	1.12 (0.92, 1.32)	0.21 (0.04, 0.37)	**0.02**	−0.22 (−0.42, −0.03)	**0.03**
MUO	1.19 (1.08, 1.29)	1.30 (1.16, 1.43)	0.11 (−0.004, 0.22)	0.06		
HDL-C (mmol/L)^c^ (*n* = 102)						
MHO	1.25 (1.17, 1.32)	1.29 (1.22, 1.37)	0.05 (−0.01, 0.11)	0.09	0.15 (0.07, 0.24)	**<0.001**
MUO	1.09 (1.04, 1.14)	1.14 (1.09, 1.20)	0.06 (0.02, 0.09)	**0.005**		
*Lipids*						
TC (mmol/L)^c^ (*n* = 102)						
MHO	4.69 (4.36, 5.02)	4.59 (4.31, 4.87)	−0.11 (−0.36, 0.15)	0.42	0.13 (−0.21, 0.47)	0.45
MUO	4.56 (4.34, 4.79)	4.47 (4.27, 4.66)	−0.09 (−0.27, 0.08)	0.29		
LDL-C (mmol/L)^c^ (*n* = 102)						
MHO	3.25 (2.90, 3.61)	3.53 (3.16, 3.89)	0.27 (0.06, 0.49)	**0.01**	−0.11 (−0.53, 0.30)	0.59
MUO	3.33 (3.09, 3.57)	3.67 (3.42, 3.92)	0.34 (0.19, 0.49)	**<0.001**		
Apo A-1 (mmol/L)^c^ (*n* = 100, missing, *n* = 2)						
MHO	186.27 (172.84, 199.70)	174.05 (162.12, 185.99)	−12.22 (−21.39, −3.05)	**0.01**	8.46 (−5.95, 22.86)	0.25
MUO	176.96 (167.67, 186.24)	166.45 (158.21, 174.71)	−10.50 (−16.84, −4.16)	**0.001**		
Apo B (mmol/L)^c^ (*n* = 100, missing, *n* = 2)						
MHO	90.48 (80.90, 100.01)	82.51 (74.15, 90.85)	−7.98 (−14.92, −1.03)	**0.03**	−10.05 (−20.13, 0.03)	0.05
MUO	100.15 (93.53, 106.77)	92.93 (87.16, 98.70)	−7.22 (−12.02, −2.42)	**0.004**		
Apo B/Apo A-1^c^ (*n* = 100, missing, *n* = 2)						
MHO	0.50 (0.45, 0.56)	0.49 (0.43, 0.55)	−0.01 (−0.05, 0.03)	0.65	−0.08 (−0.15, −0.02)	**0.02**
MUO	0.58 (0.54, 0.62)	0.58 (0.53, 0.62)	−0.003 (−0.03, 0.02)	0.83		
*Insulin resistance*						
Fasting insulin (µU/mL)^c^ (*n* = 94, missing, *n* = 8)						
MHO	16.71 (13.15, 20.27)	20.73 (15.74, 25.72)	4.02 (−1.06, 9.10)	0.12	0.01 (−4.27, 4.29)	0.99
MUO	18.12 (15.64, 20.61)	19.29 (15.81, 22.78)	1.17 (−2.37, 4.72)	0.51		
HOMA-IR^c^ (*n* = 94, missing, *n* = 8)						
MHO	3.88 (2.74, 5.03)	4.90 (3.51, 6.29)	1.02 (−0.49, 2.52)	0.18	−0.52 (−1.77, 0.73)	0.41
MUO	4.71 (3.92, 5.49)	5.12 (4.16, 6.08)	0.42 (−0.62, 1.45)	0.43		
TG/HDL-C^c^ (*n* = 102)						
MHO	0.75 (0.58, 0.92)	0.90 (0.70, 1.10)	0.15 (−0.02, 0.32)	0.08	−0.34 (−0.55, −0.13)	**0.002**
MUO	1.14 (1.02, 1.26)	1.19 (1.05, 1.33)	0.06 (−0.06, 0.17)	0.35		
QUICKI^c^ (*n* = 94, missing, *n* = 8)						
MHO	0.33 (0.31, 0.34)	0.31 (0.30, 0.33)	−0.01 (−0.03, 0.01)	0.21	0.01 (−0.01, 0.02)	0.45
MUO	0.31 (0.31, 0.32)	0.32 (0.31, 0.33)	0.00 (−0.01, 0.01)	0.79		
*Inflammatory markers*						
Uric acid (mmol/L)^c^ (*n* = 100, missing, *n* = 2)						
MHO	0.37 (0.34, 0.40)	0.34 (0.31, 0.38)	−0.03 (−0.05, −0.001)	**0.04**	−0.03 (−0.06, 0.01)	0.21
MUO	0.39 (0.37, 0.41)	0.38 (0.35, 0.40)	−0.01 (−0.03, 0.005)	0.16		
Adiponectin (µg/mL)^c^ (*n* = 97, missing, *n* = 5)						
MHO	7.27 (6.47, 8.08)	7.49 (6.55, 8.42)	0.21 (−0.88, 0.46)	0.53	1.36 (0.39, 2.34)	**0.007**
MUO	5.89 (5.33, 6.45)	6.14 (5.50, 6.78)	0.25 (−0.21, 0.71)	0.29		
hsCRP (mg/L)^c^ (*n* = 90, missing, *n* = 12)						
MHO	2.60 (1.50, 3.69)	2.15 (1.16, 3.13)	−0.45, (−1.40, 0.50)	0.35	−0.97 (−2.09, 0.16)	0.91
MUO	3.33 (2.57, 4.08)	3.35 (2.68, 4.03)	0.03 (−0.63, 0.68)	0.93		
Interleukin-6 (pg/mL)^c^ (*n* = 91, missing, *n* = 11)						
MHO	2.19 (1.73, 2.66)	3.09 (2.35, 3.83)	0.90 (0.23, 1.57)	**0.009**	−0.24 (−0.87, 0.38)	0.45
MUO	2.44 (2.13, 2.76)	3.32 (2.82, 3.83)	0.88 (0.43, 1.34)	**<0.001**		

The data is expressed as the means (standard deviation). Mean differences within groups (week-16 compared with baseline) and between groups (MHO compared with MUO) before and after intervention were analysed using repeated measures ANOVA adjusted for confounders and covariates. Bold *p* values indicate statistical significance

*MHO* metabolically healthy obesity, *MUO* metabolically unhealthy obesity, *CRFs* cardiometabolic risk factors, *BMI* body mass index, *PBF* percentage of body fat, *WC* waist circumference, *SMM* skeletal muscle mass, *PFS* physical fitness score, *SBP* systolic blood pressure, *DBP* diastolic blood pressure, *FPG* fasting plasma glucose, *TC* total cholesterol, *TG* triglycerides, *HDL-C* high-density lipoprotein, *LDL-C* low-density lipoprotein, *Apo A-1* apolipoprotein A-1, *Apo B* apolipoprotein B, *HOMA-IR* homeostasis model assessment of insulin resistance, *QUICKI* quantitative insulin sensitivity check index, *hsCRP* high-sensitivity C-reactive protein, *95% CI* 95% confidence interval, *mean diff* mean difference

^a^Adjusted for age and gender

^b^Adjusted for age, gender, and height

^c^Adjusted for pubertal status

At the end of week-16 of the intervention, 51.6% of participants in the MHO group had transitioned to the MUO status, while 26.8% of participants in the MUO group had crossed over to the MHO status (Table 3). Within the MUO group, the frequency of CRFs showed a decreasing trend except for an increased percentage of individuals with abnormal FPG and no changes in triglycerides. Additionally, the odds of having SBP > 90th percentile were 59% lower at week-16 of intervention than at baseline (OR = 0.41 (95% CI: 0.18, 0.92), *p* = 0.03) in the MUO group.

**Table 3:** Distribution of CRFs and odds ratios after the intervention

Status at baseline	Baseline	Week-16	Probability of CRFs after the intervention	
	*n* (%)	*n* (%)	OR (95% CI) (week-16, baseline)	*p* value
MUO phenotype				
MHO	0 (0.0)	16 (51.6)	–	–
MUO	71 (100)	52 (73.2)	–	–
FPG > 5.6 mmol/L				
MHO	0 (0.0)	7 (22.6)	–	–
MUO	21 (29.6)	25 (35.2)	1.29 (0.68, 2.46)	0.43
HDL-C ≤ 1.03 mmol/L				
MHO	0 (0.0)	6 (19.4)	–	–
MUO	34 (47.9)	26 (36.6)	0.63 (0.39, 1.00)	0.05
TG > 1.7 mmol/L				
MHO	0 (0.0)	3 (9.7)	–	–
MUO	12 (16.9)	12 (16.9)	1.00 (0.54, 1.86)	1.00
SBP > 90th percentile				
MHO	0 (0.0)	4 (12.9)	–	–
MUO	15 (21.1)	7 (9.9)	0.41 (0.18, 0.92)	**0.03**
DBP > 90th percentile				
MHO	0 (0.0)	6 (19.4)	–	–
MUO	26 (36.6)	19 (26.8)	0.63 (0.32, 1.26)	0.19

The data are expressed as the proportion (%) of CRFs at baseline and at week-16. The probability of having CRFs after the intervention among children with the MUO phenotype was determined using generalized estimating equations (GEEs) and is expressed as the odds ratio (OR) and 95% confidence interval (CI). Bold *p* value indicates statistical significance

*MHO* metabolically healthy obesity, *MUO* metabolically unhealthy obesity, *CRFs* cardiometabolic risk factors, *FPG* fasting plasma glucose, *HDL-C* high-density lipoprotein, *TG* triglycerides, *SBP* systolic blood pressure, *DBP* diastolic blood pressure, *OR* odds ratio, *95% CI* 95% confidence interval

## Discussion

Lifestyle modification intervention had a favourable effect on metabolic risk in children with obesity. Both MHO and MUO resulted in significant improvements in BMI, body fat percentage, SMM, and PFS following the intervention. The intervention led to improvements in SBP, DBP, and HDL-C in children with MUO, and uric acid in children with MHO. Additionally, our findings showed that MHO status in children is transient.

Our findings demonstrated a high prevalence of severe obesity and metabolically unhealthy school-aged children. Worryingly, children with severe obesity are associated with an increased prevalence of cardiometabolic risks [33], and they are more likely to remain obese in adulthood and develop obesity-related complications [34]. The American Academy of Paediatrics has recommended shifting focus to CRF clustering, which is often associated with childhood obesity, and applying the most intensive intervention effort to lower the risk [35]. Obesity management in the form of lifestyle modification during childhood has been suggested as the first-line strategy for lowering cardiometabolic risk [36]. Furthermore, several systematic reviews suggest that school-based interventions may be the most feasible and effective approach for weight loss among school-aged children [37, 38].

The observed improvements in body composition parameters and CRFs in both groups are consistent with those observed in adult studies [20], implying that lifestyle interventions could benefit both MHO and MUO, with the latter benefitting the most. However, it was also noted that the participants in the MHO group were already metabolically healthy at baseline, which might explain why the cardiometabolic parameters did not change much following the intervention. While extensive studies regarding the effect of these interventions on metabolic phenotypes in adults have been published, similar evidence in children with obesity is still limited. A recently published study reported improvements in SBP and DBP after weight loss intervention in children with MUO [39], and their results are consistent with our findings.

We found that approximately half of the children with MHO at baseline transitioned to MUO status at the end of the intervention, whereas one in four participants in the MUO group crossed over to MHO status. Although this study could not explain the exact mechanism behind this transition, several possible causes exist. One key explanation is the hormonal changes that occur during puberty. A study found that transitioning from pre- to mid-puberty causes an increase in insulin resistance, raising the likelihood of switching from metabolically healthy obesity (MHO) to metabolically unhealthy obesity (MUO). Conversely, as children progress from mid- to late puberty, insulin resistance decreases, increasing the likelihood of crossing over from MUO to MHO phenotype [40]. Another possible explanation is the genetic predisposition of each child to develop metabolic abnormalities; however, this topic is beyond the scope of this paper.

MHO in children does not necessarily translate to decreased morbidity and mortality later in adulthood [41], and evidence has shown that many of these individuals will develop MUO, thus increasing their risk of major diseases [42, 43]. Therefore, MHO is a transient state of metabolic abnormality development. Previously, we reported that more than half of children with MUO had only one risk factor, most commonly high blood pressure [21]. Our previous findings concur with a study by Yang et al. [39] indicating that MUO in children is typically mild and that the management of blood pressure is critical for preventing MUO. Indeed, our lifestyle intervention is effective at lowering the likelihood of developing high SBP.

Our intervention resulted in a significant increase of HDL-C levels, and a reduced likelihood of having low HDL-C among children with MUO although marginally not significant. In addition, both groups had significantly increased PFS and SMM. Exercise has been shown to increase HDL-C levels [44] and physical fitness [45], and to protect against cardiovascular diseases [46]. Among the non-traditional CRFs, Apo A-1 and Apo B, which play significant roles in lipid metabolism, are known to be independent predictors of ischemic heart disease [47], and a study reported that exercise significantly decreased the Apo B:Apo A-1 ratio in children with obesity [48]. Although our study showed a significant reduction in the Apo A-1 and Apo B levels in both groups after the intervention, no changes in the Apo B: Apo A-1 ratio were observed. Another study that investigated the association of MHO in children with carotid intima-media thickness (cIMT), a proxy for cardiovascular diseases, reported higher cIMT in both the MHO and MUO groups than in metabolically healthy normal-weight children [49]. This highlights the importance of weight control in children, regardless of metabolic status.

Obesity appears to cause low-grade inflammation, and adipose tissue inflammation is thought to play a role in the pathogenesis of insulin resistance and metabolic disturbance [50]. Adults with MHO had lower levels of pro-inflammatory markers such as hsCRP and IL-6 than those with MUO [51], but the association in children remains inconclusive [52, 53]. On the contrary, adiponectin, an anti-inflammatory marker, was found to be an independent predictor of MHO in children [21, 54]. A study reported a significant decrease in IL-6 and a significant increase in adiponectin following a short-term lifestyle intervention among children with obesity [55], however, we could not produce similar findings. Nevertheless, both the MHO and MUO groups in this study had significantly higher levels of IL-6 post-intervention, indicating that subclinical inflammation persists albeit adiponectin was significantly higher in the MHO group than in the MUO group. Uric acid, another non-traditional CRF and has been reported as an independent predictor of MHO in children [56, 57], was found to be significantly lower at week-16 in the MHO group in this study. In children with obesity, hyperuricaemia has been associated with an increased risk of type 2 diabetes and cardiovascular diseases [58].

Regarding insulin resistance, we did not observe significant changes in fasting insulin and HOMA-IR levels. Although the hyperinsulinemic-euglycemic clamp is regarded as the gold standard for identifying insulin resistance, due to its invasiveness and complexity, the calculation of the HOMA-IR index is mostly used as a surrogate marker of insulin resistance. However, HOMA-IR in children does not always correlate well with the gold standard [59], and the association with metabolic phenotypes in children is ambiguous [21, 52, 60–63]. Therefore, the insulin resistance consensus group did not recommend the use of fasting insulin to screen for insulin resistance [64], and further research is needed to identify a strong surrogate marker of insulin resistance [65]. On the contrary, QUICKI, another surrogate marker for insulin resistance has been reported to have a stronger correlation with the gold standard [66], nevertheless, we did not observe any changes at the end of the intervention. A large cohort study has recommended using the TG: HDL-C ratio as an inexpensive and reliable surrogate marker of insulin resistance in children with obesity [67]. Since then, research has demonstrated the utility of the TG: HDL-C ratio as an insulin resistance marker and for identifying children at risk for metabolic syndrome [68–70]. On the other hand, in our study, we observed no significant changes in the TG: HDL-C ratio after the intervention, even though the MHO group had a significantly lower value than the MUO group.

### Strengths and limitations

Despite the study’s notable findings, several limitations must be addressed. First, this study has a rather small sample size from an epidemiological standpoint and used a quasi-experimental design. Second, this study lacked information on modifiable factors such as dietary and lifestyle habits outside the school period. Finally, because our intervention lasted for only 16 weeks, longer-term randomized clinical trials are warranted to obtain more convincing results. One of the strengths of this study was that the intervention was conducted at the community level, which avoided the bias of the controlled environment. We also examined obesity-related parameters beyond traditional CRFs, such as uric acid, inflammatory markers, Apo A-1, and Apo B, to corroborate our study. Moreover, the use of the recently proposed definition of MHO in children is critical to facilitate comparison with the findings of future studies.

## Conclusion

In conclusion, our findings demonstrate that children with either MHO or MUO can benefit equally from lifestyle interventions to improve body composition, although CRFs improved more prominently in the MUO group than in the MHO group. Thus, our data support the notion of emphasizing the importance of weight control in children, regardless of metabolic status. More importantly, our findings indicate that the MHO phenotype in children is transient. Therefore, early targeted interventions such as strategies to lower blood pressure and increase HDL-C could be proven useful for protecting children against a metabolically unhealthy state.

## Data Availability

The dataset used for this study are available from the corresponding author upon reasonable request.

## Abbreviations

MHO: Metabolically healthy obesity
MUO: Metabolically unhealthy obesity
CRFs: Cardiometabolic risk factors
BMI: Body mass index
SMM: Skeletal muscle mass
PFS: Physical fitness score
FPG: Fasting plasma glucose
TG: Triglycerides
HDL-C: High density lipoprotein cholesterol
LDL-C: Low density lipoprotein cholesterol
SBP: Systolic blood pressure
DBP: Diastolic blood pressure
HOMA-IR: Homeostasis model assessment of insulin resistance
Apo A-1: Apolipoprotein A-1
Apo B: Apolipoprotein B
IL-6: Interleukin-6
hsCRP: High sensitivity C-reactive protein
cIMT: Carotid intima media thickness
OR: Odds ratio
95% CI: 95% Confidence interval
sd: Standard deviation
ANOVA: Analysis of variance
GEE: Generalized estimating equation
